# Evaluation of Orexin-A Salivary Levels and its Correlation with Attention After Non-invasive Brain Stimulation in Female Volleyball Players

**DOI:** 10.1186/s40798-024-00698-5

**Published:** 2024-04-04

**Authors:** Fiorenzo Moscatelli, Antonietta Monda, Antonietta Messina, Marcellino Monda, Vincenzo Monda, Ines Villano, Antonella De Maria, Mancini Nicola, Gabriella Marsala, Maria Ida de Stefano, Pierpaolo Limone, Giovanni Messina, Rita Polito

**Affiliations:** 1 Department of Wellbeing, Nutrition and Sport, Pegaso Telematic University, Naples, Italy; 2 Department of Human Science and Quality of Life Promotion, San Raffaele Telematic University, Rome, Italy; 3 Department of Experimental Medicine, Section of Human Physiology and Unit of Dietetics and Sports Medicine, Università degli Studi della Campania “Luigi Vanvitelli”, Naples, Italy; 4 Department of Movement Sciences and Wellbeing, University of Naples “Parthenope”, Naples, Italy; 5 Faculty of Physical Education and Sports, “Babes Bolyai” University, Cluj-Napoca, Italy; 6 Drug’s Department, ASP Catania, Catania, Italy; 7 Department of Clinical and Experimental Medicine, University of Foggia, Foggia, Italy; 8 Department of Psychology and Education, Pegaso Telematic University, Naples, Italy

**Keywords:** Salivary orexin-A, Transcranial magnetic stimulation (TMS), Non-invasive brain stimulation (NIBS), Attention, Sport, Athletes, Performances

## Abstract

**Background:**

The capacity to change attention from one area to another depending on the many environmental circumstances present is a crucial aspect of selective attention and is strictly correlated to reaction time. The cholinergic system of the basal forebrain is crucial for attentive abilities. Several inputs, particularly orexin neurons, whose cell bodies are found in the postero-lateral hypothalamus, can activate the cholinergic system. The aim of this study was to investigate if high frequencies rTMS at dorsolateral prefrontal cortex (DLPFC) in highly trained volleyball players can change Orexin-A levels, attention and reaction time. This study was a double-blinded (participant and evaluator) matched-pair experimental design. Twenty right-handed female volleyball players were recruited for the study (age 24.6 ± 2.7 years; height 177.0 ± 5.5 cm; body mass 67.5 ± 6.5 kg; BMI 21.5 ± 1.2).

**Results:**

The main finding of this study was that 10 Hz rTMS to the DLPFC seems to increase Orexin-A salivary levels and the percentage of correct answers, while decreasing RT. After rTMS, the athletes show an increase in the percentage of correct answers immediately after the end of stimulation, and also after 15 and 30 min. Moreover, the athletes show decreases in reaction time after the end of stimulation and after 15 and 30 min to the end of stimulation, while no differences were found at the end of stimulation. Finally, the athletes show significant increases in Orexin-A salivary levels after stimulation with a peak after 30’ of the end.

**Conclusion:**

The results of our study seem to indicate that there is a relationship between salivary Orexin-A levels and RT. These results could provide useful tools for modulating sports training; in fact, if confirmed, they could lead coaches to offer their athletes rTMS sessions appropriately integrated with training. In fact, alternating attention is a mental flexibility that enables people to change their point of focus and switch between tasks requiring various levels of cognition.

## Background

Sustained attention is the ability to maintain a constant behavioral response throughout continuous and repetitive activities, whereas selective attention is the capacity to maintain a behavioral or cognitive set in the face of competing or distracting stimuli [1]. Alternating attention is a mental flexibility that permits people to flip between tasks requiring varying levels of cognition and change their point of focus [2]. The capacity to change attention from one area to another depending on the many environmental circumstances present is a crucial aspect of selective attention [3, 4]. The different environmental circumstances influence the selective attention which is the process of focusing on a particular object in the environment for a specific period. Attention is a limited resource, so selective attention allows us to tune out unimportant details and focus on what matters. To specifically enable the accurate execution of challenging motor tasks, training develops lasting encoded habits within the adult neural system [5, 6]. Motor cortex in the primate brain was once thought to contain a simple map of the body's muscles. Recent evidence suggests, however, that it operates at a radically more complex level, coordinating behaviorally useful actions [5]. The mapping from cortex to muscles is not fixed, as was once thought, but instead is fluid, changing continuously on the basis of feedback, or training aimed at acquiring new skills, in a manner that could support the control of higher-order movement parameters. These findings suggest that the motor cortex participates directly in organizing and controlling the animal's behavioral repertoire. Athletes could serve as a useful model to study the effects of training on the corticospinal system excitability, because in this subjects, since it needs a high level of coordination for the accurate execution of technical skills in static and dynamic situations [7]. Changes in arousal typically deduced from brain activity data, whereas the study of attention is based on behavioral studies. Changes in arousal typically are deduced from brain activity data (EEG), whereas the study of attention is based on behavioral studies. The more evident arousal effect on EEG activity is the “desynchronization” phenomenon. It refers to the rapid shift from high-amplitude low-frequency EEG activity, typical of sleep, to low-amplitude high-frequency electroencephalographic activity, typical of wakefulness [7]. In attention regulation, orexins play a significant role likely via interactions with multiple ascending neuromodulatory systems, including dopamine neurons in the ventral midbrain, noradrenergic neurons in the locus coeruleus and the basal forebrain cholinergic system [7, 8]. The orexin/hypocretins are neuropeptides synthesized by a cluster of neurons within the postero-lateral hypothalamus that produce excitatory effects on target neurons. These neuropeptides were discovered simultaneously in the late 1990s by two different research teams. Because they appeared to be involved in the regulation of food and metabolism, one group of researchers gave these peptides the name orexins, derived from the Greek word "orexis," which means “appetite”. Due to their considerable sequence similarities with members of the glucagon/vasoactive intestinal polypeptide/secretin (incretin) family, the other group called these peptides hypocretins [9, 10].

Cholinergic basal forebrain (BF) structures are among the many brain regions to which orexin neurons have extensive connections. The orexin-producing neurons may boost not just arousal but also attention through certain neural pathways, according to various studies conducted in the past ten years. According to their findings, the basal forebrain may be an important location where these neurons work. In this work, we examine the effects of orexin-producing neurons and their projection to the BF to support the idea that the orexin system may promote attentional processing by enhancing cortical acetylcholine (Ach) release [8]. Orexins play a crucial role in the control of attention, possibly through interactions with several ascending neuromodulator systems, such as the basal forebrain cholinergic system, the locus coeruleus (LC), and dopamine neurons in the ventral midbrain. Orexin peptides affect attentional mechanisms in the BF by increasing cell activity and Ach release. Increased attentional states are associated with increased orexin neuron activity, which varies with arousal levels.

In neurons of the posterolateral hypothalamus and perifornical regions, orexin A and B were first discovered [11]. For this reason, it was thought that these neuropeptides were expressed only in the central nervous system and oversaw centrally regulating the nutritional balance by transmitting orexigenic effects after the activation of their receptors [12].

An increase in functional connectivity between the lateral hypothalamus and the dorsolateral prefrontal cortex (DLPFC) was recently discovered in addicted people processing negative emotions. Repeated transcranial magnetic stimulation (rTMS) of the DLPFC caused neuroendocrine changes in studies of depression, most likely due to its effects on the hypothalamus–pituitary–adrenal (HPA) axis [13]. The DLPFC, anterior cingulate, and inferior parietal cortex are key neural correlates of executive function and working memory. It has been determined that these and other brain areas are essential for accurate rehearsal, maintenance, or modification of information in working memory [14]. The DLPFC plays a special executive attention role in actively preserving access to stimulus representations and objectives in environments with plenty of distraction [15]. The rTMS is a neuromodulation technique that makes use of electromagnetic coils placed on the scalp to create a magnetic field that, depending on the delivery settings, either stimulates or inhibits cortical activity. There is general agreement that rTMS below 1 Hz at the motor cortex lowers cortical excitability, whereas rTMS over 5 Hz raises cerebral cortex excitability [16]. A systematic review found that high-frequency rTMS applied to the left DLPFC was most likely to result in selective cognitive improvement [17]. However, other studies show the following benefits: increased attentional control during the Stroop task, favorable effects of rTMS on attention in subjects with deficit hyperactivity disorder, decreased reaction time [18], fewer commission errors in a continuous performance test, and improved working memory [19]. Considering the connection between the hypothalamus and DLPFC and the role of Orexin-A producing excitatory effects on target neurons, the research hypothesis assumes that DLPFC rTMS at high frequencies, increasing Orexin-A levels could have positive effects on attention and consequently motor coordination. The rTMS administered to the DLPFC has been recommended in several randomized controlled studies as a viable technique to enhance cognitive function [20]. Animal studies have demonstrated that magnetic stimulation strengthens synaptic function or boosts neurogenesis to enhance cognitive capacity [21]. These rTMS-induced molecular and cellular alterations could serve as the substrate for the changes in human brain networks that have been more extensively researched [22]. Evidently, rTMS improves function in part through altering brain connections [22]. A recent published paper has shown that HF-rTMS of the DLPFC seems to improve performance in terms of homolateral coordination, with a significantly decreased resting motor threshold and motor evoked potential latency of the ipsilateral motor cortex [23]. These results are in line with previously published papers. In fact, different authors reported changes in the neurocognitive profile after HF-rTMS of the left DLPFC. Based on the connection between the hypothalamus and DLPFC and the excitatory effects of Orexin-A, our hypothesis assumes that high-frequency rTMS at DLPFC can increase Orexin-A salivary levels and improve attention and motor coordination in volleyball players [23, 24]. Alternating attention is a mental flexibility that enables people to change their point of focus and switch between tasks requiring various levels of cognition in volleyball, the ability to extract better quality information per fixation and to acquire information more effectively via peripheral vision contributes to increased performances. Our research hypothesis assumes that both the DLPFC and orexin are involved in attentional processes and, furthermore, high-frequency magnetic stimulation, having facilitatory effects on the DLPFC, could influence orexin levels. Furthermore, in team sports such as volleyball, the coordination of the individual athlete, the coordination between the athletes, and also the attention to game events are fundamental [25]. The aim of this study was to investigate the acute effects of high-frequency rTMS at DLPFC on Orexin-A salivary levels, attention, and reaction time in volleyball players.

## Methods

### Participants

Twenty right-handed (Cohen’s *d*: 0.56) highly trained [26] female volleyball players were recruited for the study (age 24.6 ± 2.7 years; height 177.0 ± 5.5 cm; body mass 67.5 ± 6.5 kg; BMI 21.5 ± 1.2). The volleyball players were members of two local team, regularly competing at national levels and undergoing a training regimen of at least five 2-h sessions^.^week^−1^ for the previous 5 years. To select the sample we used the following inclusion criteria: (1) the two volleyball teams participated in the same championship; (2) the two teams were coached by the same trainer; (3) the athletes must not have taken breaks in training at least in the three months preceding the study. The local Institutional Ethics Committee approved the study (Azienda Ospedaliera-Universitaria “Ospedali Riuniti”, Foggia, Independent Ethics Committee; protocol number that was attributed by the ethics committee: 116/CE/2011, 14/11/2011). All subjects recruited for the investigation provided both written and oral information regarding the possible risks and discomforts and were ensured that they were free to withdraw from the study at any time. Furthermore, a medical examination ascertained the absence of psychoactive or vasoactive medication assumption, and risk factors or other contraindication according to the safety and recommendation for TMS use. The subjects recruited for the study presented no contraindications and therefore no one was excluded from the procedure. During the 24 h preceding the start of the experimental procedures, the subjects recruited had to abstain from exercising and had to limit their caffeine intake. Before starting the experimentation, a session was carried out to familiarize ourselves with the tests foreseen by the experimental procedure.

### Experimental Procedure

**Fig. 1:**
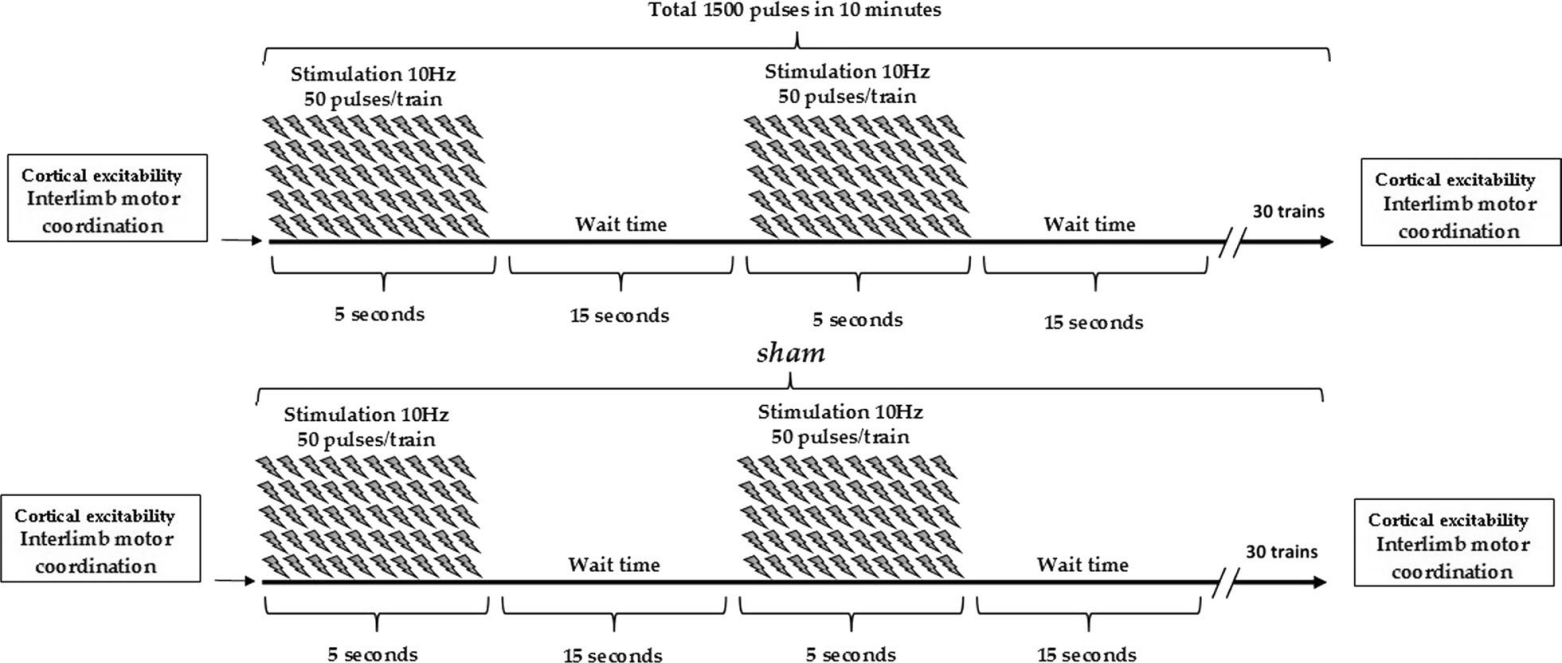
In Fig. 1 was showed the study protocol of stimulation condition (Day 1) and sham condition (Day 2). Afeter salivary samples collection and posner test (T0), the volleyball players performed rTMS at 10 Hz, 80% of the RMT of the right first dorsal interosseous muscle, 5 s of stimulation, and 25 s of rest, for a total of 1,500 pulses. Furthermore, immediately after the end of the stimulation/sham (T1), after 15' from the end of the stimulation/sham (T2) and 30′ from the end of the stimulation/sham (T3) were repeated the investigation perofromed in T0

This study was a double-blinded (participant and evaluator) matched-pair experimental design. The subjects involved in the study were sent to report to the laboratory in order to be instructed on the experimental procedure. A detailed explanation was given regarding the performance of the Posner test and the TMS procedure. The subjects recruited for the study were subjected to two experimental conditions (stimulation, sham) in two sessions 10 days apart from each other (Fig. 1). All experimental procedures were carried out in the morning. The athletes were invited to the laboratory in groups of five. The stimulation or sham stimulation was performed randomly. The sampling of salivary samples and the Posner test were performed with these times: before the TMS (T0), immediately after the end of the stimulation/sham (T1), after 15′ from the end of the stimulation/sham (T2) and 30' from the end of the stimulation/sham (T3). The order of conditions (i.e., sham and stimulation) were counterbalanced in this experimental procedure.

### TMS and Electromyographic Recording

To minimize any possible circadian influence, the recording session were performed between 9:30 AM and 12:30 AM. The experimental sessions were carried out during the rest day after the match. All tests were performed at the physiology laboratory of the University of Foggia. The temperature during the experiments was 22° and the humidity was 45%. With the subject sitting on an armchair in a quiet room, motor cortex excitability was tested using a Magstim® Rapid device (Magstim Co., Ltd., Whitland, Southwest Wales, United Kingdom) with an 80-mm figure-of-eight coil placed over the left motor cortex. A mechanical arm maintained the handle of the coil tangential to the scalp with the handle pointing backward at 45° away from the midline while delivering stimulus [23, 25]. The head of each subject was secured to the sit and throughout the procedure they remain completely relaxed. The location of the stimulation was identified on each subject’s scalp using the SofTaxic navigator system (E.M.S. Italy, http://www.emsmedical.net). The RMT was determined as the minimum stimulator intensity that evoked a peak-to-peak motor evoked potential of > 50 µV in at least 5 out of 10 consecutive trials 23, 25. Using a classical belly-tendon montage, surface pre-gelled disposable electrodes (Biopac system, snc; 1 cm, diameter) were placed in correspondence of the FDI muscle (active electrode) and over the associated joint or tendon (reference electrode), whereas the ground electrode was placed on the dorsal part of the forearm. The electrodes incorporate liquid electrolyte gel and moderately-high chloride salt concentration. The magnetic stimulator was connected to the PC, and interfaces with the EMG recording software. The stimulator sends a square wave signal (Trigger) each time it is activated. So, on the EMG trace first was shown the trigger and immediately after the muscle response.

The rTMS was delivered to the left DLPFC, which is defined as channel F3 according to the international 10–20 system. The coil was held with the handle posterior and oriented sagittally. The subjects were seated in a comfortable chair. The stimulation was performed in one session with 10 Hz, 80% [27] of the RMT of the right first dorsal interosseous muscle, 5 s of stimulation, and 15 s of rest, for a total of 1,500 pulses (Fig. 1). Sham stimulation was performed in the same manner except that the coil was held at an angle of 90*◦*, and only one edge of it rested on the scalp. During rTMS, all participants wore earplugs, and safety guidelines were followed.

### Posner Test

**Fig. 2:**
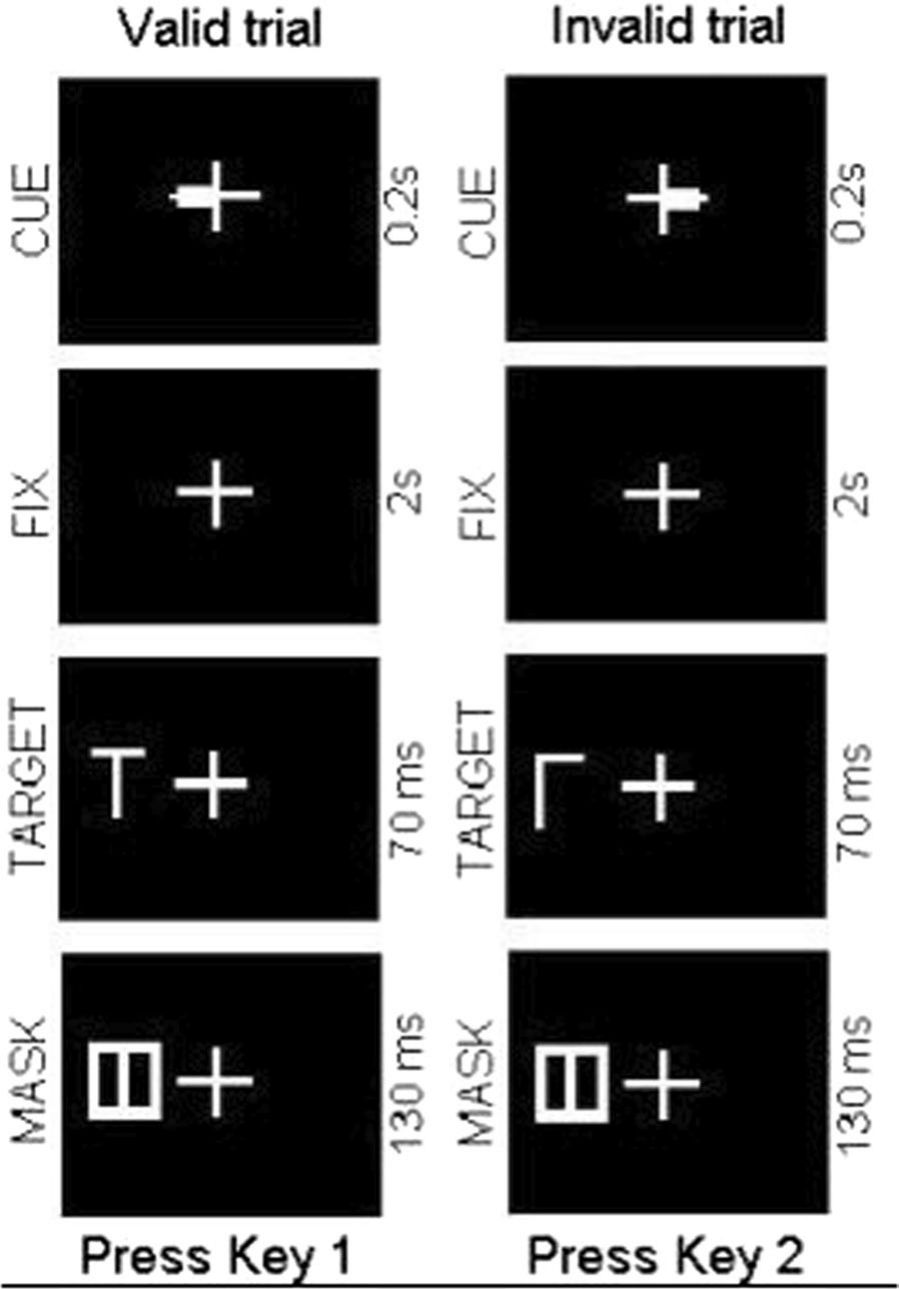
The Fig. 2 show the schematic representation of the sequences of events during a trial of Posner test

The Posner test was performed at the physiology laboratory of the University of Foggia. The Posner is a validate neuropsychological test often used to assess attention [23]. The subjects recruited for the tests were seated in a chair and placed their hands on the keyboard of a laptop. They maintained fixation on a small white cross stimulus (subtending 0.7° of visual angle) displayed on a black background in the center of a computer screen positioned at 80 cm from the nose. The trial start with the presentation of a cue stimulus (a small, white-filled rectangle subtending about 0.2° visual angle and overlapping either the left or right horizontal segment of the fixation cross) for 200 ms (ms) duration that indicated randomly (50%) either a left or right-side location along the horizontal meridian. Following a 2-s stimulus onset asynchrony, a target letter, either L or T (each with 50% probability), was presented for 70 ms at the left or right location at 0.7° degrees of visual angle from the fixation point. The letters were presented in their canonical upright orientation (50% of trials) or rotated 180 degrees along the vertical axis (the other 50%). Both letters had a diameter of 0.7° visual angle. The target stimulus appeared on 80% of the trials at the location indicated by the cue (valid trials), and on 20% of the trials at the location opposite the cue (invalid trials) [29]. Immediately after the target stimulus, a mask stimulus (130 ms duration) formed by all the possible line segments in the letter stimuli L or T was flashed to interrupt stimulus processing. Subjects were trained to remain attentive to the presentation of various stimuli on the computer screen. Also press the left keyboard button (Letter A key) when the letter T appears on the screen with the indices finger of the left hand and press the right keyboard button (Letter L key) when it compares the letter L on the PC screen with the indices finger of the right hand. The assignment of ‘target’ stimulus (T or L) to the specific key for response (A or L) was counterbalanced across subjects. This arrangement insured that the central cue did not provide any information about the response to execute, but only information about the location of the stimulus. This is important to ensure that preparatory processes were visuo-spatial in origin and not motor related. The experimental protocol included the administration of 64 trials for each subject. The RT (average reaction times of correct answers) and the accuracy of the response were recorded for behavioral analyses (Fig. 2). Before starting the test, the subjects were explained the execution of the test and were shown a short video showing the execution. The reaction time and the number of correct answers were considered.

### Orexin-A Salivary Assay

Salivary samples were collected between 09:00 and 11:00 before the TMS (T0), immediately after the end of the stimulation/sham (T1), after 15' from the end of the stimulation/sham (T2) and 30' from the end of the stimulation/sham (T3) using of cotton swabs (Salivette, Sarstedt, Rommelsdorf, Germany). Participants were asked to position the cotton swab in their mouth for a minimum of two minutes and then insert it into a special plastic tube. Samples were sent as soon as possible to the laboratory and kept at − 20 °C until the Orexin-A assay. The secretion samples were centrifuged at 1500 × *g* for 15 min at 4 °C. To gauge the absence of blood contamination, a secretion blood contamination kit was used. Orexin-A salivary concentrations were measured using commercial kits (Human OXA-Orexin A, ELISA Kit Elabscience E-EL-H1015), the optical density (OD) is measured spectrophotometrically at a wavelength of 450 nm, as per the manufacturers’ directions. All samples were tested in triplicate and analyzed in duplicate.

### Statistical Analysis

Statistical analyses were performed by the GraphPad 6 Software, Inc., for Windows, version 6.01. The number of the participant was checked used Sample Size calculator (Determine Sample Size: Confidence level 95%; Confidence Interval 22.48; Population 20; Sample Size Needed 10).

The data are presented as mean (M) ± standard deviation (SD), and statistical significance was set at *p* < 0.05. The Shapiro–Wilk test was used to check the normal distribution of variables (p value: T0 = 0.94; T1 = 0.93; T2 = 0.78; T3 = 0.95). The homogeneity Leven’s test show the following results: Orexin stimulation condition:T0 = 0.25; T1 = 0.15; T3 = 0.51; T3 = 0.54; Orexin sham condition: To = 0.07; T1 = 0.12; T2 = 0.053; T3 = 0.16; RT stimulation condition: T0 = 0.053; T1 = 0.051; T2 = 0.15; T3 = 0.078; RT sham condition: T0 = 0.052; T1 = 0.55; T2 = 0.049; T3 = 0.048; % of errors stimulation condition: T0 = 0.13; T1 = 0.59; T2 = 0.71; T3 = 0.47; % of errors sham condition: T0 = 0.10; T1 = 0.42; T2 = 0.12; T3 = 0.49.

A 2 (stimulation condition, sham condition) × 4 (time: T0, T1, T2, T3) analysis of variance were performed in order to investigate the differences in the two conditions after the end of stimulation, after 15’ of the end of stimulation and after 30’ of the end of stimulation. If the overall F test was significant, Tukey’s post-hoc comparisons were used. The overall effect size for ANOVA test was the following: Orexin stimulation condition = 2.19; Orexin sham condition = 1.23; RT stimulation condition = 2.45; RT sham condition = 0.57; % of errors stimulation condition = 1.15; % of errors sham condition = 0.98.

Finally, linear regression was performed in order to model the relationship between two variables by fitting a linear equation to observed data. One variable is considered to be an explanatory variable (Orexin-A), and the other is considered to be a dependent variable (Posner results in T3).

## Results

**Fig. 3:**
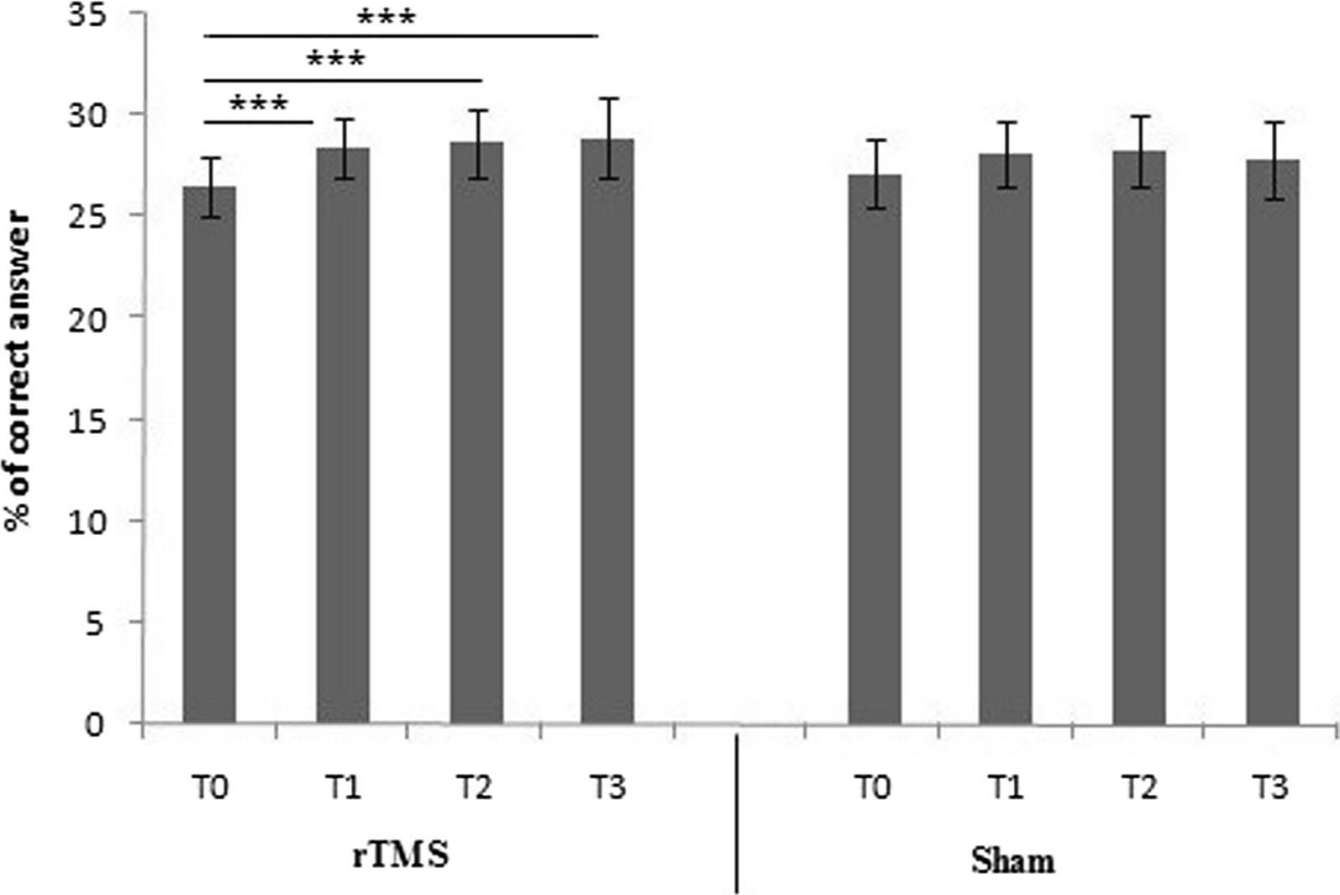
Differences in % of correct answer in stimulation condition (rTMS) and in sham condition (Sham). T0: before the TMS; T1: immediately after the end of the stimulation/sham; T2: after 15' from the end of the stimulation/sham; T3: 30' from the end of the stimulation/sham. ***p < 0.001

No discomfort or adverse effect were reported during and after TMS procedure. In active stimulation condition the percentage of correct answer during the Posner test changed from a mean value of 26.50 ± 1.46% (T0), to 28.45 ± 1.95% (T1), to 28.65 ± 1.66% (T2), to 28.90 ± 1.44% (T2). In sham condition, the percentage of correct answer during the Posner test changed from a mean value of 27.15 ± 1.63% (T0), to 28.15 ± 1.59% (T1), to 28.25 ± 1.77% (T2), to 27.89 ± 1.93% (T3). Main effects emerged for time (F = 16.7(3, 114); *p* < 0.001; ES = 0.55) with stimulation condition showing higher increase in the percentage of correct answer immediately after the end of the stimulation (T1), after 15' from the end of the stimulation (T2), and 30' from the end of the stimulation (T3) compared to sham condition. Post hoc analysis show significant differences in stimulation condition between T0 and T1 (*p* < 0.001), between T0 and T2 (*p* < 0.001) and between T0 and T3 (*p* < 0.001), while no differences emerged in sham condition (Fig. 3). No effect were found for group (F = 2.35(3, 114); *p* > 0.05, ES = 0.51).

**Fig. 4:**
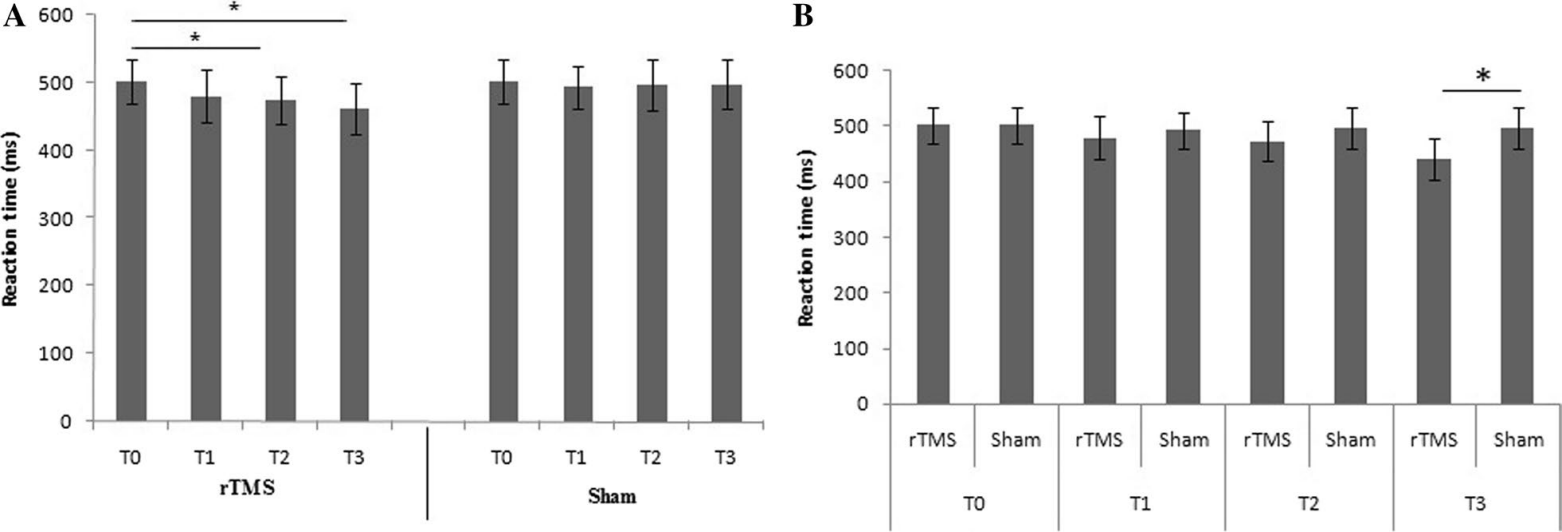
**A** Differences in reaction time (ms) in stimulation condition (rTMS) and in sham condition (Sham). T0: before the TMS; T1: immediately after the end of the stimulation/sham; T2: after 15′ from the end of the stimulation/sham; T3: 30' from the end of the stimulation/sham. *p < 0.05; ms = milliseconds. **B** Differences in reaction time (ms) between group condition; post hoc analysis show significant differences also after 30’ minute of the end of rTMS (T3); *p < 0.05; ms = milliseconds

In active stimulation condition the RT during the Posner test changed from a mean value of 502. ± 31 ms (T0), to 479 ± 38 ms (T1), to 473 ± 37 ms (T2), to 461 ± 35 ms (T3) (Fig. 2). In sham condition, the percentage of correct answer during the Posner test changed from a mean of value 502 ± 28 ms (T0), to 493 ± 32 ms (T1), to 496 ± 36 ms (T2), to 497 ± 36 ms (T3). Main effect emerged for time (F = 4.39(3, 114); *p* < 0.01; E.S. = 0.48) with stimulation condition showing significant decrease in the reaction time after 15' from the end of the stimulation (T2), and 30' from the end of the stimulation (T3) compared to sham condition. Post hoc analysis show significant differences in stimulation condition between T0 and T2 (*p* < 0.05) and between T0 and T3 (*p* < 0.05), while no differences emerged in sham condition (Fig. 4A). Main effect emerged for group (F = 4.22(3, 114); *p* < 0.05; ES = 0.42) with stimulation condition showing significant differences between group also 30’ of the end of stimulation (T3) (Fig. 4B).

**Fig. 5:**
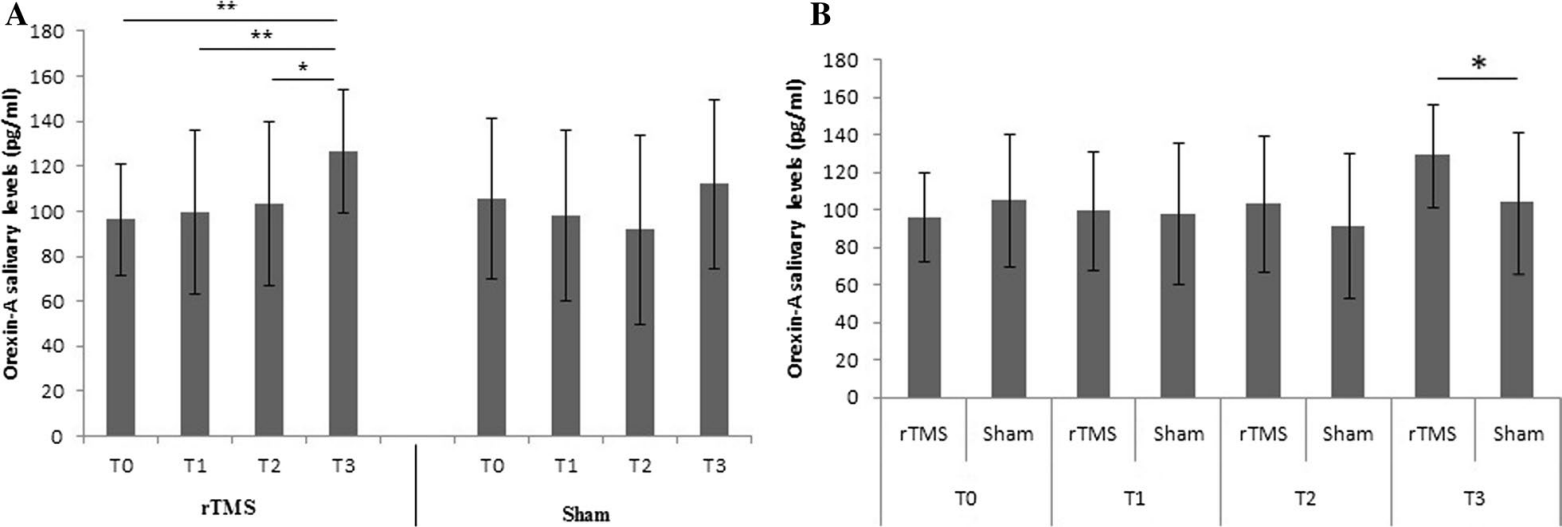
**A** Differences in Orexin-A salivary level (pg/ml) in stimulation condition (rTMS) and in sham condition (Sham). T0: before the TMS; T1: immediately after the end of the stimulation/sham; T2: after 15′ from the end of the stimulation/sham; T3: 30′ from the end of the stimulation/sham. *p < 0.05; **p < 0.01. Differences in Orexin-A salivary level (pg/ml) between group. post hoc analysis show significant differences also after 30′ min of the end of rTMS (T3); *p < 0.05

In active stimulation condition Orexin-A salivary level changed from a mean value of 96.41 ± 24.79 pg/ml (T0), to 99.39 ± 36.49 pg/ml (T1), to 103.40 ± 36.40 pg/ml (T2), to 126.47 ± 27.40 pg/ml (T3) (Fig. 3). In sham condition, Orexin-A salivary level changed from a mean value 105.38 ± 35.78 pg/ml (T0), to 98.21 ± 37.84 pg/ml (T1), to 91.66 ± 42.07 pg/ml (T2), to 112.01 ± 37.77 pg/ml (T3) Main effect emerged for time (F = 3.19(3, 114); *p* < 0.001;E.S. = 0.67) with stimulation condition showing significant increase in the Orexin-A salivary levels after 30' from the end of the stimulation (T3) compared to sham condition. Post hoc analysis show significant differences in stimulation condition between T0 and T3 (*p* < 0.01) and between T1 and T3 (*p* < 0.01), and between T2 and T3 (*p* < 0.05), while no differences emerged in sham condition (Fig. 5A). Main effect emerged for group (F = 3.98(3, 114); *p* < 0.05; ES = 0.49) with stimulation condition showing significant differences between group also 30’ of the end of stimulation (T3) (Fig. 5B).

**Fig. 6:**
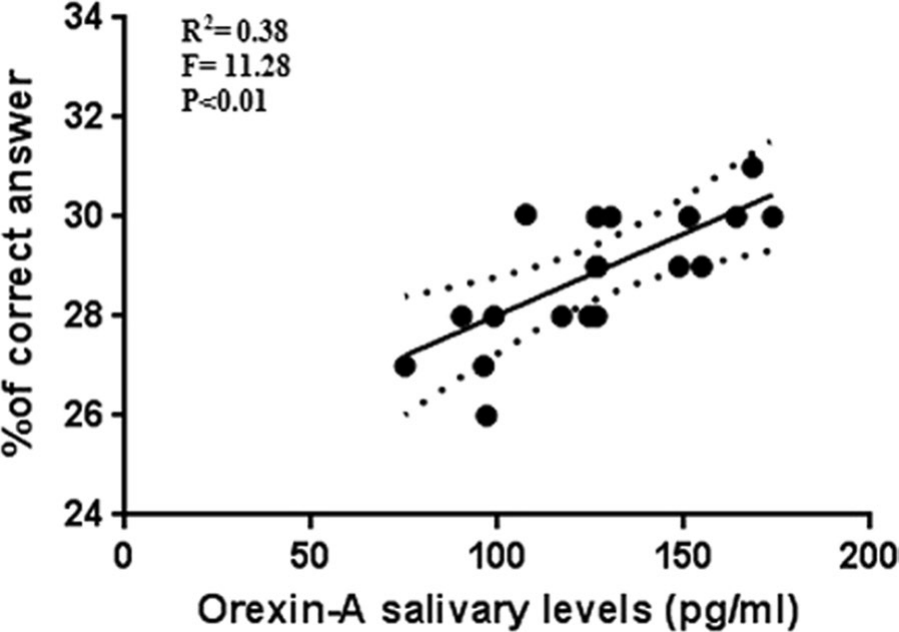
Relationship between Orexin-A and the percentage of correct answer

**Fig. 7:**
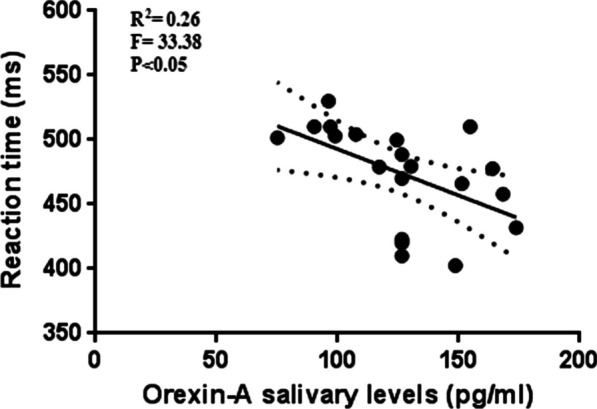
Relationship between Orexin-A and the reaction time

We observed the highest levels of Orexin 30' after the end of the stimulation session, therefore we carried out a regression analysis with the parameters measured with the Posner test in order to verify the presence of any relationships. In active stimulation condition, after 30’ of the end of stimulation (T3), the athletes show the higher number of correct answer, the best reaction time and the higher levels of salivary Orexin-A (Fig. 3), thus we have performed linear regression analyses to investigate the relationship between these parameters. The results show significant relationship between Orexin-A and the percentage of correct answer at T3 (Fig. 6) and significant relationship between Orexin-A and reaction time at T3 (Fig. 7).

## Discussion

The main finding of this study was that 10 Hz rTMS to the DLPFC seems to increase Orexin-A salivary levels and the percentage of correct answers, while decreases RT. After rTMS, the athletes show an increase in the percentage of correct answers immediately after the end of stimulation, and also after 15 and 30 min. Moreover, the athletes show decreases in reaction time after the end of stimulation and after 15 and 30 min to the end of stimulation, while no differences were found at the end of stimulation. Finally, the athletes show significant increases in Orexin-A salivary levels after stimulation with a peak after 30′ of the end. Furthermore, regression analysis showed significant relationships between orexin levels and correct response rates and between orexin levels and reaction times. To the best of our knowledge, this is the first study that has investigated the modulation of salivary Orexin-A levels using rTMS, the percentage of errors and reaction time in athletes. Despite the fact that, at baseline, the athletes show a good Orexin-A salivary levels, as we also expected from the literature data [24], in active stimulation conditions, after 30’ of the end of stimulation, the athletes show increased levels of salivary Orexin-A, a higher number of correct answers and the best reaction time and linear regression analysis shows a relationship between salivary Orexin-A levels and the Posner test. These results seem to show that HF rTMS could modify Orexin-A levels, attention, and motor responses.

Recent study shown that Brain slice patch clamp recordings from orexin neurons show that they can intrinsically generate tonic firing in a regular, pacemaker-like manner. In vitro, this intrinsic activity can be slowly modulated by specific nutrients, gasses, and neuromodulators. However, the activity dynamics of orexin neurons in vivo change much more rapidly than in brain slices, likely reflecting the brain-wide neural inputs that they receive [27]. Recent orexin neural network imaging at cellular resolution indicates that the rapid dynamics of orexin cells during wakefulness appears to be a property of most orexin cells. 2-photon calcium imaging of > 300 orexin neurons during locomotion reveals that the majority (around 70%) of orexin neurons activate around initiation of running bouts. Optogenetic evidence indicates that this peri-initiation activity of orexin cells appears to be causally linked to locomotion initiation. Optogenetic excitation of orexin cells at frequencies resembling their natural in vivo firing, produces frequency-dependent running [28]. In turn, optogenetic inhibition of orexin neurons makes both sensory-evoked and self-paced running less likely [28]. These experiments supply causal evidence for a role of orexin neurons in rapid sensorimotor control in the awake brain [28]. The finding that subsecond sensory dynamics of orexin cells produces rapid locomotor control, which is not entirely dissimilar to cortical-mediated sensorimotor transformations, clarifies why orexin cells may need to update their awake activity on a subsecond timescale.

Studies have also focused on the neuropeptide dynorphin, which is created in orexin neurons and has distinct effects on different classes of BF neurons, to better understand how orexin-producing neurons increase cortical activation. Although Orexin-A directly excites cholinergic neurons, which do not respond to dynorphin, there are two additional groups of non-cholinergic basal forebrain neurons. One of these groups of non-cholinergic sleep-promoting neurons is activated by Orexin-A and does not respond to dynorphin; the other population is repressed by dynorphin but does not respond to Orx-A [25]. To promote attention and enhance cognitive function, the co-release of orexins and dynorphin can activate a synergistic mechanism that activates cholinergic and non-cholinergic wake-active neurons and suppresses non-cholinergic sleep-active neurons. These results provide significant evidence in favor of the theory that orexin stimulation of the BF can enhance cortical activation and attention by acting on cholinergic and non-cholinergic neurons in response to salient stimuli. Orexins stimulate cholinergic neurons; as a result, the rise in acetylcholine release from the cerebral cortex plays a role in the cortical activation related to attention. Positive and negative feedback processes that are mediated by the lateral hypothalamus/perifornical region govern orexin neurons. Orexins form a positive feedback loop that depolarizes orexin neurons, opens nonselective cation channels, and regulates presynaptic glutamate release [8]. By activating astrocytes with glutamate and causing them to discharge lactate and protons into the extracellular space using monocarboxylate transporters, glutamatergic transmissions indirectly excite orexin neurons [29, 30]. Orexin neurons also employ astrocyte-derived lactate as an energy source to support physical activity. Moreover, the release of protons owing to monocarboxylate transporter activity results in a temporary drop in extracellular pH, which can induce orexin neurons to depolarize [31].

A recent investigation revealed that orexin neurons fire more frequently during active wakefulness, less frequently as animals approach sleep, and stop firing altogether during slow-wave and REM sleep. Therefore, given that transcranial high-frequency resistance has an excitatory effect, this could affect orexin levels and positively affect attentional and decision-making processes [32]. Regarding the physiology of HF-rTMS over DLPFC, prior research indicated that a single session of HF-rTMS caused the ipsilateral head of the caudate nucleus in the striatum to release dopamine. The dopamine neurons in the striatum were affected by the DLPFC stimulation because it improved the efficiency of the glutamate neurotransmitter and glutamate receptors. Additionally, some glutamate synaptically connected with medium spiny neurons that were almost in the ventral tegmental region (VTA) via their dendritic spines. This process aided in controlling the release of dopamine in the VTA because of the stimulation of dopamine neurons in the region. Therefore, HF-rTMS of the DLPFC may result in an enhancement in visuospatial processing and consequently, an improvement in motor performance [23].

Therefore, an increase in orexin levels could induce excitatory effects and improve attention. These effects are very important when it comes to sports performance; in fact, reducing reaction times and improving attention lead to more effective motor responses which, therefore, improve sports performance. So, it seems that HF rTMS of the DLPFC is able, by increasing the orexin level, to positively influence some parameters of sports performance. In daily life, and particularly in sport, we constantly witness the control of actions and motor responses to ensure that they are suitable for multiple motor actions [33–36]. These situations reflect the so-called "perception–action cycle", introduced by Fuster (2003), which highlights the continuous interaction of perceptual neural networks with the executive component, presented by the motor cortex, which, interacting with each other, can perform the movements correctly [33]. As previously described, one of the most important consequences of these relationships is the ability to make a response that is appropriate to the perceived stimulus [2, 35, 36].

Our results are in line with previously published papers. In fact, different studies reported changes in the neurocognitive profile after high-frequency rTMS on the left DLPFC [25]. Authors recently reported that, HF rTMS to the DLPFC, significantly improved working memory, performance and RT [12]. Determining how rTMS-induced neurobiological alterations impact cognitive performance is difficult [37–39]. According to the accumulating data, rTMS induces alterations in interconnected areas as well as neurophysiological and neurochemical changes in the area that is stimulated [22, 40–42]. The activation of a neural network that supports the target functions or the suppression of a rival network that hinders the target functions can both improve cognition [43–47]. However, despite the interesting results, the study has some limitations that should be investigated in the future to clarify all aspects. It would be advisable to repeat the study by increasing the sample size. The results of our study show a main effect for the group both for the orexin and for the RT, even if the post hocs show a slight significance only in T3, instead as regards the number of correct answers, no effect is evident principal for the group. Furthermore, a main effect for time emerged for both orexin and RT and for the number of correct answers; in this case the post hoc analysis shows significant differences between the times. These results show that there may be interactions between rTMS and these parameters, however they should be investigated further for confirmation.

However, we would like to underline that this is partly due to the fact that we have taken into consideration two teams of volleyball who play in the same series to avoid distortions. Study subjects included only younger female volleyball players, and in the future it could be interesting to investigate also men volleyball players in order to exclude hormonal changes factors. Furthermore, the effects of rTMS at different frequencies should also be evaluated (in our study we performed rTMS only at 10 Hz as used in previus published study [23] to establish the best protocol to achieve performance improvement. Furthermore, a study including both male athletes and non-athletes should be conducted. Moreover, this study's evidence has not been compared to that of the general population, and thi is an aspct to investigate in the future, in order to understand if these results are specific to volleyball players or even females or for all population. Finally, it is also worth considering that the tests performed by the volleyball players were not very related to the volleyball tasks and the results may not carry over.

## Conclusions

Our study shows that a single session of HF rTMS on DLPFC in volleyball players appears to increase salivary Orexin-A levels (from 96.41 ± 24.79 before stimulation to 126.47 ± 27.40 pg/ml after 30’ of stimulation); furthermore improvement in RT emerged in T3. The results of our study seem to indicate that there is a relationship between salivary Orexin-A levels and RT. These results could provide useful tools for modulating sports training; in fact, if confirmed, could lead coaches to offer their athletes rTMS sessions appropriately integrated with training. Alternating attention is a mental flexibility that enables people to change their point of focus and switch between tasks requiring various levels of cognition. Responding to numerous tasks or various task demands simultaneously is referred to as divided attention. Despite the limitations described at the end of discussion section, we believe that these results could be of great interest to the scientific community and could have practical implications in the future. In consideration of the importance of orexin, and its implications, we believe that our study can provide the starting point for carrying out new investigations in this area.

## Data Availability

All data were entered into the manuscript.

## Abbreviations

BF: Basal forebrain
HF: High frequencies
DLPFC: Dorsolateral prefrontal cortex
BMI: Body mass index
TMS: Transcranial magnetic stimulation
NIBS: Non-invasive brain stimulation
Ach: Acetylcholine
LC: Locus coeruleus
HPA: Hypotalamus-pituitary-adrenal
rTMS: Repetitive transcranial magnetic stimulation
RT: Reaction time
VTA: Ventral tegmental region
FDI: First dorsal interosseus
